# Validation of a Novel Strategy for Fluorescence Quenching for a Self-Quenching Fluorogenic Probe and Its Application for Visual Loop-Mediated Isothermal Amplification Detection During Food Safety Analysis

**DOI:** 10.3390/foods13233816

**Published:** 2024-11-26

**Authors:** Sisi Huang, Shihui Wang, Tianlong Wang, Hongwei Song, Yan Guo, Xiong Xiong, Libin Wang

**Affiliations:** 1 College of Food Science and Light Industry, Nanjing Tech University, Nanjing 211816, China; 202121019089@njtech.edu.cn (S.H.); wsh0326@njtech.edu.cn (S.W.); wtl@njtech.edu.cn (T.W.); shw@njtech.edu.cn (H.S.); 202461119020@njtech.edu.cn (Y.G.); 2 College of Light Industry and Food Engineering, Nanjing Forestry University, Nanjing 210037, China; wanglibin2013@sina.cn

**Keywords:** loop-mediated isothermal amplification, self-quenching fluorogenic probe, visual inspection, sequence-specific detection, on-site detection

## Abstract

The self-quenching fluorogenic probe facilitates precise identification of LAMP (loop-mediated isothermal amplification) amplicons, unaffected by non-specific products resulting from primer dimers. However, low quenching efficiency by surrounding nucleobases leads to high background signal, posing significant challenges for visual inspection with the naked eye. The present study aims to identify an oligonucleotide sequence that is complementary to the self-quenching fluorogenic probe, and to employ the fluorescence super-quenching mechanism of double-stranded DNA to establish a visualization system for the LAMP assay. The results indicated that the incorporation of a sequence fully complementary to the probe could significantly reduce the system’s background fluorescence (*p* < 0.05). When the melting temperature exceeds room temperature, truncating the complementary sequence from the 3′ end does not compromise the probe’s quenching efficiency. The LAMP visualization system, using a 10–13-base complementary sequence of the loop primer-based probe, could effectively minimize background fluorescence and yield straightforward visual results post-reaction. Applied to rainbow trout and Atlantic salmon detection, the system detected 1 pg DNA in a closed-tube format. In conclusion, a suitable complementary sequence can reduce the background fluorescence of the self-quenching fluorogenic probe. Employing this sequence alongside the self-quenching fluorogenic probe to develop a low-background fluorescence LAMP system demonstrates great potential for successful visual detection and holds considerable promotional merit.

## 1. Introduction

Loop-mediated isothermal amplification (LAMP) is an isothermal nucleic acid amplification technique, first disclosed by Notomi et al. in 2000 [1]. This technology utilizes a design with four specific primers on six regions of the target, and uses Bst DNA polymerase with strand displacement activity to carry out the reaction under isothermal conditions. The initial denaturation step and possible temperature changes throughout the reaction in a traditional PCR assay thus become unnecessary. Positive samples can be swiftly identified, either by visual inspection of rising turbidity or color change, or through agarose gel electrophoresis, which reveals a ladder-like pattern indicative of DNA concatemers [2,3,4]. Due to the advantages of rapid detection, simple operation, high sensitivity, and on-site convenience, LAMP technology has, since its public disclosure, found extensive application in food adulteration detection [5,6], human epidemic detection [7], plant disease detection [8], and other fields due to its rapid, efficient, and highly specific nature.

The LAMP reaction necessitates the utilization of multiple primer pairs which may readily interact and form dimeric structures when concentrated within the system. Moreover, primer dimers would also occur when primers bind to each other instead of the target template, resulting in homodimers (formed by two identical primers) and heterodimers (formed by two primers with complementary sequences) [9]. These primer dimers can interfere with amplification and lead to nonspecific amplification, potentially causing false-positive results due to the similarity of primer sequences. Furthermore, LAMP, which uses multiple long primers at high concentrations, is particularly prone to primer dimer formation. The use of 4–6 primers in LAMP increases the likelihood of primer–primer hybridization, facilitating dimer formation. Additionally, the long primers (such as FIP and BIP) in LAMP can easily form secondary structures like hairpins, which can interfere with a primer’s annealing to the target template and lead to nonspecific amplification [2]. Furthermore, high concentrations of magnesium ions, deoxynucleotide triphosphate (dNTP), and DNA polymerase can also increase the likelihood of dimer formation. Therefore, primer dimers are formed during the LAMP amplification process due to the characteristics of the primers and various other factors, generating nontarget products and causing nonspecific amplification [10]. The situation could become even worse, since traditional methods, including fluorescent dyes like SYBR Green I and SYTO 9, as well as colorimetric indicators such as hydroxylnaphthol blue, calcein, and cresol red, are unable to distinguish false positives arising from nonspecific amplification, which can result in the incorrect interpretation of results [11]. Therefore, it makes sense to develop a LAMP detection system which would be much more specific.

The sequence-specific LAMP method, leveraging the target-specific probes or modified primers as biological recognition elements to determine the results, can accurately detect amplicons and is not affected by non-specific products [9,11]. Currently, probes designed for LAMP application include the assimilated probe [12], fluorescence bidirectional displacement probe [13], self-quenching fluorogenic probe [14], and molecular beacon [15]. Among them, the self-quenching fluorogenic probe, characterized by its simplicity in design and high sensitivity, has garnered increasing attention in LAMP, having proven its feasibility and functionality [16]. Specifically, the self-quenching fluorogenic probe is an oligonucleotide sequence that has been modified by the inclusion of a fluorescent group at the second or third position of the 3′ end of either the inner primer or the loop primer. Within this sequence, bases with low oxidation potentials undergo photoinduced electron transfer, which would result in the fluorescence quenching only when the probe is unbound to its target [14]. However, the use of a self-quenching fluorogenic probe in LAMP detection is primarily limited by their inability to visually distinguish the fluorescence states of positive and negative reactions [17]. Consequently, their application mainly involves real-time fluorescence monitoring or integration with other technologies to achieve visualization. For instance, Gadkar et al. [14] designed a loop primer serving as a self-quenching fluorogenic probe for detecting varicella-zoster virus using the real-time fluorescence curve. In terms of visual detection, Li et al. [18] combined a self-quenching fluorogenic probe with polyethylenimine (PEI), and discerned the outcome based on the formation or absence of a fluorescent precipitate after the reaction. Moreover, Nazarenko et al. [19,20] introduced a fluorescently labeled strand with a structure similar to that of a self-quenching fluorogenic probe and designed a fluorescent primer with a blunt-ended hairpin structure that utilizes complementary sequences to quench fluorescence. In this way, the visualization of multiple polymerase chain reactions (PCR) was successfully achieved.

This study delves into the realm of self-quenching fluorogenic probes, with the exploration of various labeling types serving as the primary research focus. By constructing complementary sequences with distinct structural characteristics, the study compares the quenching efficiency of these sequences on the probe, aiming to illuminate the signal response mechanism underpinned by these complementary sequences. Furthermore, the study establishes a LAMP system with a significantly reduced background fluorescence, which serves as a pivotal step towards achieving LAMP visualization detection utilizing self-quenching fluorogenic probes. This innovative approach has the potential to facilitate rapid species identification of Atlantic salmon (*Salmo salar*) and rainbow trout (*Oncorhynchus mykiss*), thereby enhancing the efficiency and accuracy of species identification in the aquatic market.

## 2. Materials and Methods

### 2.1. Probe and Complementary Sequences Designing

Five different self-quenching fluorogenic probes were obtained from the previous research of our research group, including BIP-FAM, ZJ-FAM, S-LB-6-FAM, R-FIP-FAM, and R-LB-FAM, targeting Atlantic cod (*Gadus morhua*), skipjack tuna (*Katsuwonus pelamis*), Atlantic salmon (*S. salar*), and rainbow trout (*O. mykiss*). These probes can all be applied to LAMP amplification of the corresponding species, meeting the requirements of real-time fluorescence-based LAMP detection. At the same time, the fully complementary sequences were obtained by reverse complementation based on the base arrangement order of the self-quenched probes. Partially complementary sequences were designed by introducing mismatched bases, extending the 5′ end, shortening the 5′ end, and shortening the 3’ end on the fully complementary sequences. The sequences are summarized in detail in Table 1.

The fluorescence intensity of the self-quenching fluorogenic probes and the probe-complementary sequence duplex were monitored in relation to increasing temperature, ranging from 35 °C to 97 °C, at a frequency of 10 times per degree centigrade, using the Gentier 96E real time PCR system (Tianlong, Xi’an, China). In particular, the fluorescence intensity at 35 °C for each probe and duplex was recorded for further analysis.

### 2.2. Real-Time LAMP Reaction

The LAMP assay was conducted in real time, with a total reaction volume of 20 μL. The reaction mixture was optimized by varying the concentrations of the inner primer (0.6 μM), each of the loop primers (0.4 μM), the self-quenching fluorogenic probe (0.4 μM), and the complementary sequence (0.4 μM), MgSO_4_ (1.0–3.5 mM), dNTPs mix (Takara Biomedical, Beijing, China) (0.45 mM). In all reactions, 0.2 μM of each outer primer, 2.5 μL 10× ThermoPol buffer, 8 units of Bst DNA polymerase (Vazyme Biotech, Nanjing, China), and 50 ng of DNA template were used.

The amplification process was carried out in a Gentier 96E real time PCR system, under the following conditions: 64 °C for 60 min (one cycle per min). Fluorescence signals were recorded at the conclusion of each cycle, and successful amplification was indicated by a sigmoid-shaped fluorescence curve.

### 2.3. Endpoint Visual LAMP Reaction

For the visual LAMP assay, the reaction mixture (20 μL) was similar to the one used in the previously described optimized real-time assay. The reaction took place in a metal bath (AoSheng, Hangzhou, China) at a temperature of 64 °C, for a specified duration. Once the reaction was complete, a blue gel cutter (Uelandy, Suzhou, China) with an excitation wavelength of 470 nm was used to capture the fluorescence image, with the reaction tubes being placed directly on it. Positive results were identified by the presence of a bright green fluorescence visible to the naked eye, while weaker green fluorescence was observed in blank and non-target samples.

### 2.4. Statistical Analysis

All statistical analyses were carried out using SPSS Statistics software version 27 (SPSS Inc., Chicago, IL, USA). The results were all presented as mean ± standard error. Duncan’s multiple range test was used to compare differences among the means. Differences were considered statistically significant at a level of 5% (*p* < 0.05).

## 3. Results and Discussion

### 3.1. Strategy for Accurate Screening of the Complementary Sequences

The self-quenching fluorogenic probe, leveraging the photoinduced electron transfer mechanism, holds immense promise for sequence-specific detection in the LAMP reaction [16,17]. Nevertheless, its high background signal poses a significant challenge in visualizing the LAMP reaction’s endpoint [21]. According to a previous study [22], the intensity of the fluorescence signal from these probes is critically influenced by the complementary sequences, making the identification and screening of the optimal complementary sequence a pivotal aspect of this research. In this study, the self-quenching fluorogenic probe (coded as BIP-FAM) targeted the inner primer of Atlantic cod (*G. morhua*), which was selected as a case study. A series of adjustments were made to the fully complementary sequence of the probe, as detailed in Table 1. These modifications included: (1) introducing single mismatch at the modification site of the fully complementary sequence, (2) truncating the fully complementary sequence at both the 3′ and 5′ ends, and (3) extending the fully complementary sequence at the 5′ end. Upon hybridization with the self-quenching fluorogenic probe, the fluorescence intensity at 35 °C was compared for each complementary sequence.

**Figure 1:**
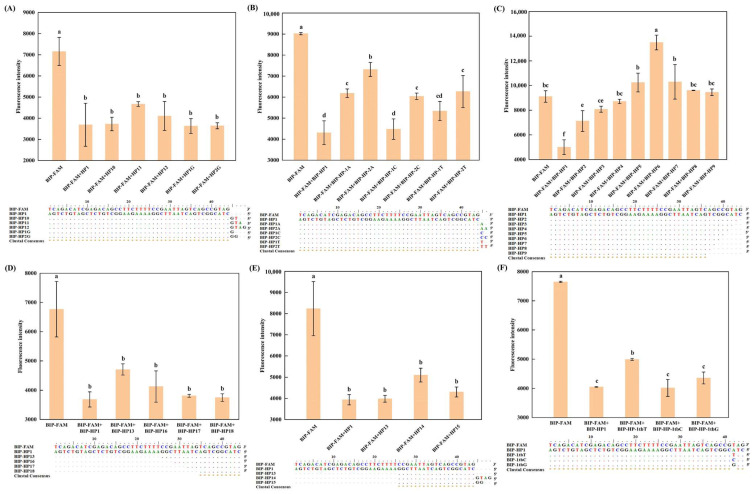
Effects of the complementary sequences in different statuses on the fluorescence intensity of the self-quenching fluorogenic probe. (**A**) represents the fully complementary sequences sequentially added with GTAG and GG from the 5′ end; (**B**) represents the fully complementary sequences with one or two A, T, C added from the 5′ end; (**C**) represents the fully complementary sequences with a gradual nucleotide reduction from the 5′ end; (**D**) represents the fully complementary sequences with a gradual nucleotide reduction from the 3′ end; (**E**) represents the partially complementary sequences added with GTAG and GG from the 5′ end; (**F**) represents the fully complementary sequences with one mismatch at the fluorophore modification site. For each bar graph, different letters indicate significant differences in terms of fluorescence intensity (*p* < 0.05).

As illustrated in Figure 1B, when either one or two A, T, and C bases were added to the 5′ end of the complementary sequence, respectively, a significant increase in the fluorescence intensity was observed (*p* < 0.05) for the extended complementary sequence hybridized with the self-quenching fluorogenic probe. Furthermore, the complementary sequence extended by two nucleotides exhibited a higher fluorescence intensity compared to the one extended by just one nucleotide upon hybridization. One plausible explanation for this observation could be that an increase in the number of bases in the non-complementary region boosts the overall rigidity of the DNA strand, impeding electron transfer between the DNA double strands and consequently reducing the quenching effect of the complementary sequences on the self-quenching fluorogenic probe [23,24]. However, when a G base was added to the 5′ end of the complementary sequence for extension, the fluorescence intensity of the hybridized extended sequence did not exhibit a significant change (*p* > 0.05) when bound to the self-quenching fluorogenic probe (Figure 1A,E). The G base-mediated fluorescence quenching is consistent with previous study [25].

Additionally, the shortened complementary sequence can also affect the fluorescence intensity of the self-quenching fluorogenic probe. Specifically, Figure 1C demonstrates an elevated background signal when employing a fully complementary sequence truncated from the 5′ end. This may be attributed to enhanced quenching due to electron transport within the double-stranded DNA, which is more efficient than in the single-stranded state [23]. Conversely, truncating the complementary sequence from the 3′ end to 10 nucleotides does not impair the quenching effect (Figure 1D). Additionally, the mismatches introduced into the complementary sequence of the probe’s labeling site are also unable to enhance the quenching effect, and when the base at the mismatch site is replaced, changing from A to T, the quenching efficiency is instead undermined (Figure 1F). Therefore, an appropriate complementary sequence can effectively minimize the background fluorescence of the self-quenching fluorogenic probe. Integrating this complementary sequence with the self-quenching fluorogenic probe creates, significantly, the possibility of establishing a LAMP system with low-background fluorescence, demonstrating the system’s applicability in visual detection methods.

### 3.2. Optimization of the Complementary Sequences

**Figure 2:**
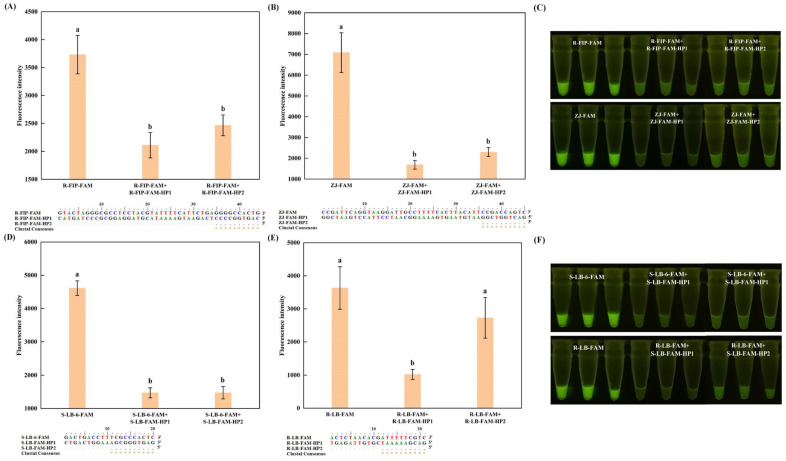
Effects of the complementary sequences shortened from the 3′ end on the fluorescence intensity of the self-quenching fluorogenic probe. R-FIP-FAM, ZJ-FAM, S-LB-6-FAM, and R-LB-FAM are the self-quenching fluorogenic probes. For each probe, HP1 represents the fully complementary sequence; HP2 represents the 10-nucleotide-length complementary sequence. (**A**,**E**) represent the results for rainbow trout (*O. mykiss*), with the probe designed based on inner primer and loop primer, respectively; (**B**) represents the results for skipjack tuna (*K. pelamis*); and (**D**) represents the results for Atlantic salmon (*S. salar*). (**C**,**F**) represent the values for the visual inspection under UV light. Different letters on the bar graph indicate significant differences (*p* < 0.05).

Although a fully complementary sequence, even with several additional G bases at the 5′ end, can achieve a satisfactory quenching effect, an excessively long complementary sequence poses a risk of disrupting the reaction’s normal progression [21]. Furthermore, as illustrated in Figure 1D, no significant difference (*p* > 0.05) in fluorescence intensity was observed when using complementary sequences ranging from 44 to 10 nucleotides. Consequently, we opted to design complementary sequences with lengths of 10 nucleotides for the four previously studied self-quenching fluorogenic probes. Upon hybridization with the self-quenching fluorogenic probe, changes in the fluorescence intensity were recorded and compared. The results indicated that a 10-nucleotide complementary sequence could generate the same quenching effect as a fully complementary sequence for R-FIP-FAM, ZJ-FAM, and S-LB-6-FAM (Figure 2A–D,F). However, for R-LB-FAM, due to its low Tm value (R-LB-FAM-HP2, 27.6 °C), the hybridization with the probe could be disrupted during heating, leading to a poor quenching effect and thus, to a high background signal (Figure 2E,F).

**Figure 3:**
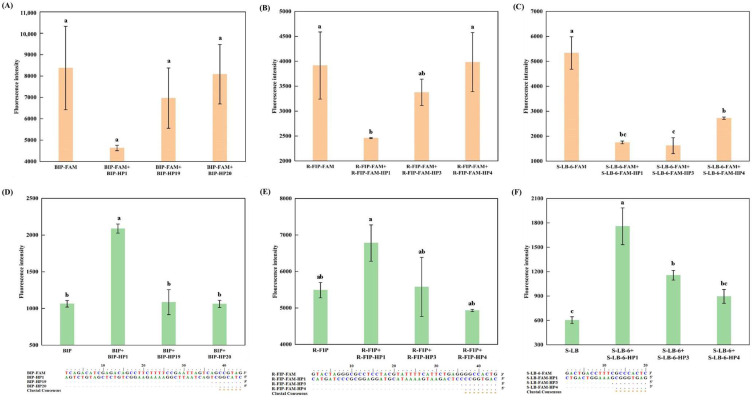
Effects of the shortened complementary sequence on the fluorescence intensity of the self-quenching fluorogenic probe and the unlabeled primer. BIP-FAM, R-FIP-FAM, and S-LB-6-FAM are the self-quenching fluorogenic probes, with the corresponding primer coded as BIP, R-FIP, and S-LB, respectively. For each probe, HP n (n = 1, 3, 4, 19, 20) represents the complementary sequence (fully or partially). (**A**,**D**) represent the results for Atlantic cod (*G. morhua*), with the probe-based and SYTO 9-based assays, respectively; (**B**,**E**) represent the results for rainbow trout (*O. mykiss*), with the probe-based and SYTO 9-based assays, respectively; and (**C**,**F**) represents Atlantic salmon (*S. salar*), with the probe-based and SYTO 9-based assays, respectively. Different letters on the bar graph indicate significant differences (*p* < 0.05).

**Figure 4:**
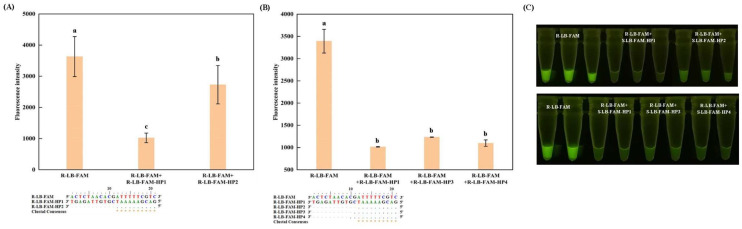
Effects of the complementary sequences shortened from the 3′ end on the fluorescence intensity of the self-quenching fluorogenic probe R-LB-FAM. HP n (n = 1, 2, 3, 4) represents the complementary sequence (fully or partially). (**A**,**B**) represents the fluorescence intensity according to the melting curve. (**C**) represents the visual inspection under UV light. Different letters on the bar graph indicate significant differences (*p* < 0.05).

To further validate the significance of the Tm value, in conjunction with the complementary sequence, as to probe quenching, an additional study was conducted on three self-quenching fluorogenic probes using complementary sequences of varying lengths (Figure 3). As the length of the complementary sequence decreased, the stability of hybridization between the sequence and the unlabeled probe fragment diminished, which became evident from the gradual weakening of the fluorescence intensity (Figure 3D–F). Instead, the fluorescence intensity of the complementary sequences hybridized with the self-quenching fluorogenic probes and increased significantly (Figure 3A–C). Inspired by these findings, we conducted a gradient experiment on the length of the complementary sequence for R-LB-FAM and found that a complementary sequence 12 nucleotides in length could achieve a satisfactory fluorescence quenching effect comparable with that of the fully complementary sequence (Figure 4). These results are also consistent with a previous study [26].

**Figure 5:**
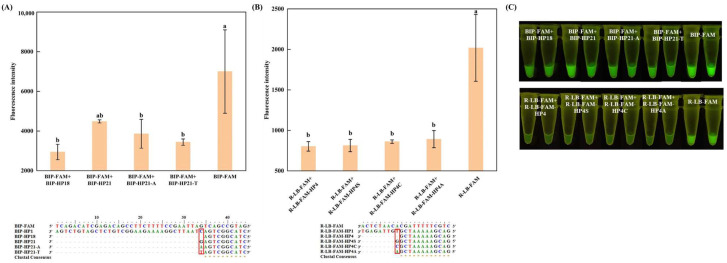
Effects of the single mismatch from the 3′ end of the complementary sequence on the fluorescence intensity of the self-quenching fluorogenic probe. BIP-FAM, and R-LB-FAM are the self-quenching fluorogenic probes. For each probe, HP n (n = 1, 4, 18, 21, 4S, 4C, 4A, 21-A, 21-T) represents the complementary sequence (fully or partially). The mismatch from the 3′ end of the complementary sequence was highlighted using red rectangle. (**A**) represents the results for Atlantic cod (*G. morhua*). (**B**) represents the results for rainbow trout (*O. mykiss*). (**C**) represents the visual inspection under UV light. Different letters on the bar graph indicate significant differences (*p* < 0.05).

**Figure 6:**
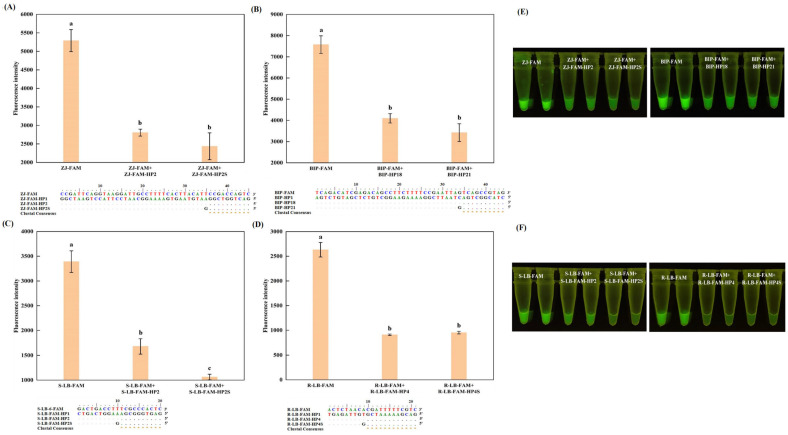
Effects of the G mismatch from the 3′ end of the complementary sequence on the fluorescence intensity of the self-quenching fluorogenic probe. ZJ-FAM, BIP-FAM, S-LB-6-FAM, and R-LB-FAM are the self-quenching fluorogenic probes. For each probe, HP n (n = 1, 2, 2S, 4, 4S, 18, 21) represents the complementary sequence (fully or partially). (**A**) represents the results for skipjack tuna (*K. pelamis*); (**B**) represents the results for Atlantic cod (*G. morhua*); (**C**) represents the results for Atlantic salmon (*S. salar*); and (**D**) represents the results for rainbow trout (*O. mykiss*). (**E**,**F**) represent the visual inspection under UV light. Different letters on the bar graph indicate significant differences (*p* < 0.05).

Although the optimal length of the complementary sequence has been established, directly incorporating it into the reaction system is impractical due to the potential extension by DNA polymerase [27]. A common strategy to circumvent this issue is to introduce additional mismatch from the 3′ end at the complementary sequence [21]. As illustrated in Figure 5B, no significant difference was observed for the introduced mismatch in terms of the fluorescence intensity (*p* < 0.05), and given the enhanced quenching effect of G base [25], R-LB-FAM-HP4S was ultimately selected for R-LB-FAM. A similar strategy was also applied on other self-quenching fluorogenic probes, with S-LB-6-FAM exhibiting comparable performance as well (Figure 6C,D). However, for BIP-FAM and ZJ-FAM (Figure 5A and Figure 6A,B), although significant decreases in the fluorescence intensity could consistently be observed according to the real-time melting curves, visual inspection revealed a discernible background. This is in agreement with our previous finding [22], and can be attributed to the intrinsic signal of these probes, suggesting that further refinements in primer selection, or prioritizing the selection of loop primers for probe design, are necessary for improvement. Therefore, while probes designed based on inner primers can achieve fluorescence super-quenching via hybridization with complementary sequences, the optimization process for these probes tends to be more intricate and challenging. In contrast, probes designed using loop primers offer a more straightforward path to achieving a high signal-to-noise ratio for visual detection, making them a more favorable choice for simplifying the detection process while maintaining high accuracy.

### 3.3. LAMP Reaction Based on the Self-Quenching Fluorogenic Probe

**Figure 7:**
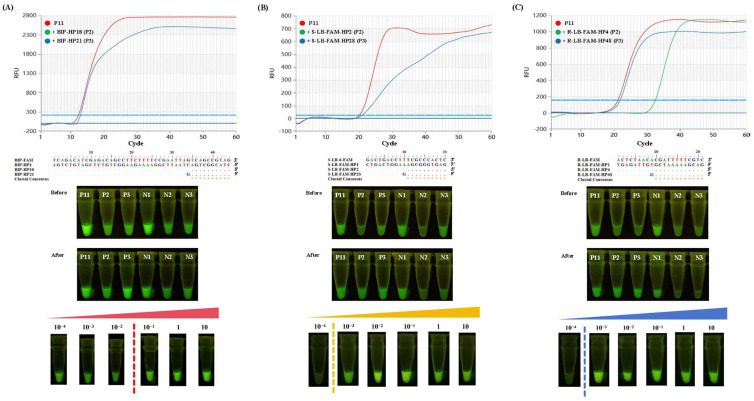
Real-time LAMP amplification and visual inspection. (**A**) represents the results for Atlantic cod (*G. morhua*); (**B**) represents the results for Atlantic salmon (*S. salar*); and (**C**) represents the results for rainbow trout (*O. mykiss*). For each species, P11 (red line) represents the LAMP reaction based on the self-quenching probe only (BIP-FAM, S-LB-6-FAM, R-LB-FAM); P2 (green line) represents the LAMP reaction based on the self-quenching probe and a partially complementary sequence (BIP-HP18, S-LB-6-FAM-HP2, R-LB-FAM-HP4); and P3 (blue line) represents the LAMP reaction based on the self-quenching probe and a partially complementary sequence modified with an additional G mismatch from the 3′ end (BIP-FAM21, S-LB-6-FAM-HP2S, R-LB-FAM-HP4S). N1-N3 represent the negative control for each amplification. For sensitivity evaluation, the 10-fold serial dilutions of total DNA (from 10 ng to 10^−4^ ng) were prepared for each species.

During the LAMP reaction, all of the reagents and primers, in addition to the probe and the complementary sequence, were pre-incorporated into the reaction system. This eliminated the need to open the lid during the process, significantly reducing the risk of cross-contamination. As shown in the real-time curves (Figure 7), all of the positives were successfully amplified, and no false positives could be observed, highlighting the specificity attained using the probe-based method. This is in agreement with previous studies [14,18]. In Figure 7C, amplification was also highlighted with R-LB-FAM-HP4, which was inconsistent with the results of the other two probes. It is speculated that the lower Tm value of R-LB-FAM-HP4 makes the hybridization complex of probe and complementary sequence open at the beginning of the reaction, thus triggering a series of amplification reactions [26]. Visual inspection confirmed the feasibility of R-LB-FAM and S-LB-6-FAM (Figure 7). Alternatively, the poor quenching effect of BIP-FAM highlighted the necessity for a primer re-design to achieve visual inspection.

With the established visual LAMP assay for BIP-FAM, S-LB-6-FAM, and R-LB-FAM, the sensitivity was determined by the minimum amount of DNA that could produce a detectable signal. In this case, 10-fold serial dilutions of genomic DNA, from 10 ng/μL to 10^−4^ ng/μL, were conducted, and the lowest quantity of the detectable DNA matched up to 0.1 ng, 1 pg, and 1 pg for the visual LAMP assay for BIP-FAM, S-LB-6-FAM, and R-LB-FAM, respectively (Figure 7). The exceptional sensitivity in the present study for S-LB-6-FAM and R-LB-FAM is comparable with that in the previous PEI-dsDNA assay [18], and much better than the one based on cresol red [28]. The high background signal is considered to be the main reason for the low sensitivity for *G. morhua*, which could be ameliorated by introducing a novel reaction vessel [22]. Therefore, with the optimized self-quenching probe, a visual LAMP assay can be successfully applied in fish species identification, achieving excellent sensitivity.

## 4. Conclusions

This study focuses on the self-quenching fluorogenic probe, screening for complementary sequences that effectively decrease the probe’s background fluorescence and applying these sequences to facilitate LAMP visualization detection. The findings revealed that a complementary sequence with a truncated 3′ end and exhibiting a Tm value higher than room temperature significantly diminished the fluorescence of the self-quenching fluorogenic probe. Furthermore, the incorporation of this complementary sequence into the LAMP system reduced the system’s background fluorescence without compromising the amplification reaction. The rapid LAMP visualization method established in this study, grounded in the use of self-quenching fluorogenic probes, allows for immediate visual results upon reaction completion, thereby simplifying the LAMP detection process and presenting promising applications for rapid on-site detection. Finally, the optimized visual LAMP assay was also validated on fish species identification, and the minimum amount of genomic DNA reached 1 pg for both rainbow trout and Atlantic salmon.

## Figures and Tables

**Table 1:** Oligonucleotide sequence information.

Code	Sequence (5′–3′)	Role Interpretation	Reference
S-LB-6-FAM	GACTGACCTTTCGCCCAC**T**C	The probe	[18]
R-LB-FAM	ACTCTAACACGATTTTTCG**T**C		
R-FIP-FAM	GTACTAGGGCGCCTCCTACGTATTTTCATTCTGAGGGGCCAC**T**G	The probe	[21]
R-FIP-FAM-HP1	CAGTGGCCCCTCAGAATGAAAATACGTAGGAGGCGCCCTAGTAC	Fully complementary sequence	
R-FIP-FAM-HP2	CAGTGGCCCC	Partially complementary sequence	
ZJ-FAM	CCGATTCAGGTAAGGATTGCCTTTTCACTTACATTCCGACCAG**T**C	The probe	[17]
BIP-FAM	TCAGACATCGAGACAGCCTTCTTTTCCGAATTAGTCAGCCG**T**AG	The probe	[22]
BIP-HP1	CTACGGCTGACTAATTCGGAAAAGAAGGCTGTCTCGATGTCTGA	Partially complementary sequence shortened from the 5’ end	
BIP-HP2	TACGGCTGACTAATTCGGAAAAGAAGGCTGTCTCGATGTCTGA		
BIP-HP3	ACGGCTGACTAATTCGGAAAAGAAGGCTGTCTCGATGTCTGA		
BIP-HP4	CGGCTGACTAATTCGGAAAAGAAGGCTGTCTCGATGTCTGA		
BIP-HP5	GGCTGACTAATTCGGAAAAGAAGGCTGTCTCGATGTCTGA		
BIP-HP6	GCTGACTAATTCGGAAAAGAAGGCTGTCTCGATGTCTGA		
BIP-HP7	CTGACTAATTCGGAAAAGAAGGCTGTCTCGATGTCTGA		
BIP-HP8	TGACTAATTCGGAAAAGAAGGCTGTCTCGATGTCTGA		
BIP-HP9	GACTAATTCGGAAAAGAAGGCTGTCTCGATGTCTGA		
BIP-HP-1tbC	CTCCGGCTGACTAATTCGGAAAAGAAGGCTGTCTCGATGTCTGA	Fully complementary sequence with single mismatch (red color)	The present study
BIP-HP-1tbG	CTGCGGCTGACTAATTCGGAAAAGAAGGCTGTCTCGATGTCTGA		
BIP-HP-1tbT	CTTCGGCTGACTAATTCGGAAAAGAAGGCTGTCTCGATGTCTGA		
BIP-HP-1A	ACTACGGCTGACTAATTCGGAAAAGAAGGCTGTCTCGATGTCTGA	Fully complementary sequence with unpaired bases at the 5’ end (red color)	
BIP-HP-2A	AACTACGGCTGACTAATTCGGAAAAGAAGGCTGTCTCGATGTCTGA		
BIP-HP-1T	TCTACGGCTGACTAATTCGGAAAAGAAGGCTGTCTCGATGTCTGA		
BIP-HP-2T	TTCTACGGCTGACTAATTCGGAAAAGAAGGCTGTCTCGATGTCTGA		
BIP-HP-1C	CCTACGGCTGACTAATTCGGAAAAGAAGGCTGTCTCGATGTCTGA		
BIP-HP-2C	CCCTACGGCTGACTAATTCGGAAAAGAAGGCTGTCTCGATGTCTGA		
BIP-HP-1G	GCTACGGCTGACTAATTCGGAAAAGAAGGCTGTCTCGATGTCTGA		
BIP-HP-2G	GGCTACGGCTGACTAATTCGGAAAAGAAGGCTGTCTCGATGTCTGA		
BIP-HP13	CTACGGCTGACTAATTCGG	Partially complementary sequence shortened from the 3’ end	
BIP-HP16	CTACGGCTGACTAATT		
BIP-HP17	CTACGGCTGACTA		
BIP-HP18	CTACGGCTGA		
BIP-HP19	TCGGCATC	Partially complementary sequence modified at the 5’ end	
BIP-HP20	GGCATC		
BIP-HP21	GAGTCGGCATC		
BIP-HP21-T	TAGTCGGCATC		
BIP-HP21-A	AAGTCGGCATC		
ZJ-FAM-HP1	GACTGGTCGGAATGTAAGTGAAAAGGCAATCCTTACCTGAATCGG	Fully complementary sequence	
ZJ-FAM-HP2	GACTGGTCGG	Partially complementary sequence modified from the 3’ end	
ZJ-FAM-HP2S	GACTGGTCGGG		
S-LB-FAM-HP1	GAGTGGGCGAAAGGTCAGTC	Fully complementary sequence	
S-LB-FAM-HP2	GAGTGGGCGA	Partially complementary sequence modified from the 3’ end	
S-LB-FAM-HP2S	GAGCGGGTGAG		
S-LB-FAM-HP3	GAGCGGGTGAA		
S-LB-FAM-HP4	GAGCGGGTGAAA		
R-LB-FAM-HP1	GACGAAAAATCGTGTTAGAGT	Fully complementary sequence	
R-LB-FAM-HP2	GACGAAAAAT	Partially complementary sequence modified from both ends	
R-LB-FAM-HP3	GACGAAAAATC		
R-LB-FAM-HP4	GACGAAAAATCG		
R-LB-FAM-HP4S	GACGAAAAATCGG		
R-LB-FAM-HP4C	GACGAAAAATCGC		
R-LB-FAM-HP4A	GACGAAAAATCGA		

**T** denotes the site for FAM attachment. The grey background was used to avoid confusion between different lines. Some words were left- or right-aligned, with the aim of facilitating the analysis of sequence differences.

## Data Availability

The original contributions presented in the study are included in the article, further inquiries can be directed to the corresponding author.
